# Randomised clinical study of plaque removal efficacy of an electric toothbrush in primary and mixed dentition

**DOI:** 10.1111/ipd.12753

**Published:** 2021-02-14

**Authors:** Esti Davidovich, Renzo A. Ccahuana‐Vasquez, Hans Timm, Julie Grender, Avi Zini

**Affiliations:** ^1^ Faculty of Dental Medicine, Hebrew University & Hadassah Jerusalem Israel; ^2^ Procter & Gamble Service GmbH Kronberg Germany; ^3^ Procter & Gamble Company Mason OH USA

**Keywords:** children, electric toothbrush, manual toothbrush, paediatric, plaque removal

## Abstract

**Background:**

Clinical investigations of electric toothbrushes in young children are limited.

**Aim:**

To assess plaque reduction efficacy of an oscillating‐rotating electric *versus* manual toothbrush in a paediatric population in primary and mixed dentitions.

**Design:**

In this randomised, single‐brushing, 2‐treatment, 4‐period, replicate‐use crossover study, subjects were divided into 2 age groups (3‐6 years; 7‐9 years) and assigned to a treatment sequence involving an Oral‐B Kids electric brush and a manual brush control. Plaque was assessed pre‐ and post‐brushing (Turesky Modified Quigley‐Hein Plaque Index). Parents brushed the teeth of their children aged 3‐6 years, whereas children aged 7‐9 years brushed their own teeth under supervision. Plaque removal scores were analysed for brush differences in each age group separately using an analysis of covariance for crossover design.

**Results:**

Forty‐one children (n = 20, 3‐6 years; n = 21, 7‐9 years) completed the study. For the primary dentition in children 3‐6 years, the electric brush reduced 32.3% more plaque than the manual brush (*P* = .005). For the mixed dentition in children 7‐9 years, the electric brush reduced 51.9% more plaque than the manual brush (*P* < .001).

**Conclusions:**

An electric toothbrush reduced significantly more plaque than a manual toothbrush in 2 paediatric age groups.

**Why this paper is important to paediatric dentists**

- Children often lack the manual dexterity, responsibility, and cooperation required to achieve optimal oral hygiene. This study adds to the limited body of research evaluating electric toothbrush use in children and demonstrates the superior plaque removal efficacy of an oscillating‐rotating electric brush relative to a manual brush in the primary dentition of children whose parents brushed their teeth and in the mixed dentition of children who brushed their own teeth.

## 1 INTRODUCTION

Despite being largely preventable, dental caries remains a significant health problem among children in both developing and industrialised nations.^1^, ^2^ Children with caries have an increased risk of developing subsequent caries in both the primary and permanent dentitions,^3^, ^4^ underscoring the importance of promoting thorough plaque removal early in life. Abundant evidence in adult populations demonstrates the superior plaque removal efficacy of electric toothbrushes compared with manual brushes.^5^, ^6^, ^7^, ^8^, ^9^ Although toothbrushing research among children is limited, Davidovich et al recently published a systematic review and meta‐analysis which showed electric toothbrushes were more effective at removing plaque than manual toothbrushes in a paediatric population.^10^Among electric toothbrushes, there is strong support for the oscillating‐rotating (O‐R) technology among adults.^6^ O‐R toothbrushes, characterised by a brush head that oscillates and rotates to remove plaque, have been shown to reduce more plaque compared with both manual and sonic electric brushes.^5^, ^7^

Limited research has evaluated the use and potential benefits of specific electric toothbrush technologies in children and, in particular, in very young children who lack the manual dexterity and responsibility to adequately brush their own teeth.^11^ Parents may not effectively remove plaque while brushing the teeth of their children due to a number of challenges, such as lack of awareness of proper brushing technique, child collaboration or difficulty manoeuvering a manual brush in the mouth of a young child.^12^

Given the lack of investigation focused on the potential benefits of electric toothbrushes in young children, the aim of this study was to assess the plaque reduction efficacy of an O‐R electric toothbrush compared with that of a regular manual brush in a paediatric population aged 3‐9 years.

## 2 MATERIALS AND METHODS

### 2.1 Subjects and study design

This randomised, single‐brushing, examiner‐blind, 2‐treatment, 4‐period, replicate‐use crossover study compared the plaque removal efficacy of an electric toothbrush and a manual toothbrush. The study was conducted between May and June 2019 in a paediatric population aged 3 to 9 years in Tel Aviv, Israel. To provide a comprehensive evaluation of the most common paediatric brushing scenarios, subjects were divided into two age cohorts; a 3‐ to 6‐year‐old cohort with parental brushing and a 7‐ to 9‐year‐old cohort self‐brushing. Eligible children were in good general health, showed evidence of dental plaque accumulation, and possessed a minimum of 16 natural teeth with facial and lingual scorable surfaces. Children who had fixed orthodontic appliances, required urgent dental treatment, had any condition that could interfere with study participation, had used antibiotics within 2 weeks prior to study initiation, and/or had received a dental prophylaxis within 1 month prior to study initiation were excluded. All children were required to present for each study visit with a parent or legal guardian.

Enrolled subjects were not permitted to participate in other oral care studies, receive elective dentistry including prophylaxis, or use antibiotics or anti‐inflammatory medications during the study period. Subjects in violation of pre‐visit food/drink and oral hygiene restrictions or who developed any condition expected to interfere with participation were subject to exclusion from the data analysis or the study. The subject consent form and the study protocol were reviewed by and approved prior to study inception by the Hadassah Medical Organisation Helsinki Committee (0240‐19‐HMO), and both the children and their parent/guardian were required to provide written informed consent before enrolling. The study was registered in the ISRCTN database (ISRCTN10901742).

#### 2.1.1 Screening (Visit 1)

A medical and dental history assessment was performed for each potential subject. Children meeting all eligibility requirements received an oral examination. Within each of the 2 age groups, subjects were randomly assigned to 1 of 4 treatment sequences—ABBA, BAAB, AABB, and BBAA, where A and B represent the study toothbrushes—according to a computer‐generated randomisation plan prepared by the sponsor in advance of study execution. Each subject ultimately used both the electric and the manual brush twice during the course of the 4‐period study.

Subjects and their parents were given the electric toothbrush and instructed to use it at home for the next 3 days (morning and evening) for familiarisation. Subjects were instructed to switch back to their regular oral hygiene products for the 3 days prior to their next visit.

#### 2.1.2 Period 1 (Visit 2)

Approximately 1 week (±3 days) after the Screening Visit, subjects and their parent(s) or legal guardian(s) returned to the study site and continuance criteria were assessed and recorded. Subjects were instructed to refrain from brushing their teeth after their morning brushing prior to the visit and to refrain from eating, chewing gum, or drinking for 3 hours prior to the visit (small sips of water were allowed up to 45 minutes prior to the visit). Subjects received an oral examination. A dental plaque disclosing solution (Mira‐2‐Ton; Hager & Werken, Germany) was applied with cotton swabs on all teeth. A clinical examiner conducted pre‐brushing plaque examinations using the Turesky Modified Quigley‐Hein Plaque Index (TMQHPI).^13^, ^14^

Subjects and their parents proceeded to a protected area to ensure examiner blinding to subject treatment assignments and test product identity. Those initially assigned to the electric brush group were given an Oral‐B Kids oscillating‐rotating electric toothbrush (D100 kids handle with EB10 brush head; Procter & Gamble, Cincinnati, OH, United States), whereas children initially assigned to the manual brush group were given a Paro Junior Soft (#742, Esro AG, 8802 Kilchberg, Switzerland) regular manual toothbrush. Parents brushed the teeth of their children in the 3‐ to 6‐year age group, and children in the 7‐ to 9‐year age group brushed their own teeth. All subjects/parents were given detailed verbal and written brushing instructions. The marketed dentifrice (Oral‐B Stages; 500 ppm sodium fluoride; Procter & Gamble, Cincinnati, OH, United States) was dispensed by clinic staff on a tongue depressor. Subjects/parents brushed their (child's) teeth using the treatment products under supervision, unaided by a mirror. Parents were provided with red safety glasses which prevented them from seeing the disclosed plaque. Children/parents using the electric brush were directed to brush according to manufacturer's instructions, whereas those assigned to the manual brush were instructed to brush their (child's) teeth in their customary manner. All subjects rinsed with water after brushing.

Subjects received post‐brushing oral examinations and the disclosing solution was applied on all teeth to stain any remaining plaque. Next, subjects received post‐brushing TMQHPI plaque examinations. Subjects were instructed to continue their regular oral hygiene products and routines at home between visits.

#### 2.1.3 Periods 2‐4 (Visits 3‐5)

Following washout periods of ≥48 hours (desired range, 2 to 7 days) between treatment period visits, subjects returned for each of the Period 2‐4 visits. At each visit, continuing eligibility was assessed. Subjects followed the same series of oral examinations, pre‐ and post‐brushing TMQHPI examinations, and supervised brushing procedures using the electric or manual toothbrush according to their assigned treatment sequence.

### 2.2 Dental plaque evaluations

The same blinded examiner evaluated TMQHPI for each subject at each visit. With the TMQHPI, disclosed plaque is scored using a 0‐5 scale on six sites per tooth (mesiofacial, facial, distofacial, mesiolingual, lingual and distolingual). Buccal, lingual, and whole mouth average plaque scores were calculated for each subject and tooth surfaces at each examination by totalling the individual plaque scores and dividing that sum by the number of gradable sites examined.

### 2.3 Statistical analysis

Pre‐brushing to post‐brushing plaque reduction for the primary dentition of the younger age group was the primary objective; plaque reduction for the mixed dentition of the older age group was a secondary objective. If the younger children had any permanent teeth, they were excluded from the analysis. A sample size calculation based on previous research using a similar study design^15^ indicated 20 subjects completing in a 2‐treatment 4‐period crossover study would give a two‐tailed alpha = 0.1 with at least 80% power to detect a difference between treatments of at least 0.144 for mixed dentition and at least 0.141 for primary dentition. TMQHPI across the whole mouth (excluding any permanent teeth for the 3‐6 year old group) was the primary variable. Plaque scores were averaged on a per‐subject basis so that each subject had a single average pre‐brushing (baseline) plaque score and another average plaque score following brushing in each of the 4 treatment periods. The difference (pre‐brushing minus post‐brushing) in average plaque scores was calculated for each subject in each treatment period.

The difference scores were analysed for treatment group differences using a mixed model analysis of covariance for a crossover design with potential terms in the model for subject (random effect), treatment, period, carryover, average pre‐brushing plaque score as the covariate and pre‐brushing plaque by treatment interaction. All statistical analyses were carried out separately for each age group for the whole mouth scores as well as for each sub‐region: molars, interproximal, buccal and lingual surfaces.

To assess for potential carryover effects for the primary endpoint of plaque difference, a statistical model was employed on the average pre‐brushing plaque scores to determine if carryover should be included in the final model. This test included the following factors: subject (random), treatment, period, and carryover. Since the carryover term was not significant at the 0.1 level (*P* > .1) the final crossover model (on the plaque reduction scores) did not include the carryover term for either age group analysis. Additionally, the pre‐brushing plaque by treatment interaction was not significant at the 0.1 level for all 7‐ to 9‐year‐old analyses or for the lingual surfaces of the 3‐ to 6‐year‐old analysis, so it was removed from the final models; however, the interaction remained in the statistical model for the whole mouth and other sub‐regions of the 3‐ to 6‐year‐old analyses. The 90% confidence intervals for each of the paired treatment differences were also computed from the final models for each age group.

The adjusted mean plaque removal scores for each treatment were analysed for statistical significance from 0 using a t‐test on the adjusted treatment mean score differences from the analysis of covariance using the final model. Treatment comparisons were 2‐sided tests carried out at the 10% significance level.

Adverse events (AEs) reported during the study were documented on AE electronic case report forms.

## 3 RESULTS

### 3.1 Subject disposition and demographics

A total of 42 paediatric subjects (20 subjects in the 3‐ to 6‐year age group and 22 subjects in the 7‐ to 9‐year age group) were enrolled and randomised separately to 1 of the 4 treatment sequences. One subject in the 7‐ to 9‐year age group discontinued after Period 1 because the child did not wish to continue using the test product, resulting in 41 subjects (97.6%) completing the trial. One subject in the 3‐ to 6 year age group mistakenly brushed her own teeth in Period 1 instead of her parents, and that period's data were declared not evaluable.

In the 3‐ to 6‐year age group, the mean subject age was 4.4 years (range, 3‐5 years) with 14 (70%) female. The mean subject age in the 7‐ to 9‐year age group was 7.8 years (range, 7‐9 years) with 10 (46%) female. All subjects (100%) were Caucasian in both groups.

### 3.2 Dental plaque evaluations

Summary statistics for the primary dentition in the younger age group and for the mixed dentition in the older age group were calculated at baseline (pre‐brushing) and following single brushing. Baseline mean TMQHPI scores were balanced across all treatment groups (*P* ≥ .233; Table 1, Table 2). In the 3‐ to 6‐year age group, mean baseline scores for the primary dentition were 3.113 and 3.165 for the electric and manual brush groups, respectively (*P* = .341). In the 7‐ to 9‐year age group, mean baseline scores for the mixed dentition were 3.315 and 3.259 (electric and manual brush groups, respectively; *P* = .233).

**Table 1:** Mean TMQHPI plaque reduction results: primary dentition

3‐6 Years of Age						
Treatment	n	Baseline mean^a^	Adjusted mean plaque reduction (SE)^b^	Treatment difference (SE)	Treatment difference *P*‐value [90% CI]^c^	Treatment difference (%)^d^
Oral‐B Kids Electric	20	3.113	0.811 (0.0504)	−0.198 (0.0669)	0.005 (−0.310 to 0.086)	32.3%
Paro Junior Soft Manual	20	3.165	0.613 (0.0497)			

^a^ Electric and manual brushes did not differ with respect to their baseline (pre‐brushing) plaque levels ( 2‐sided *P*‐value = 0.341).

^b^ Both electric and manual brushes showed a statistically significant post‐brushing *versus* pre‐brushing plaque reduction when compared to zero (Oral‐B Kids electric brush, 25.8% reduction in plaque from baseline [*P* < .001]; Paro Junior manual brush, 19.5% reduction in plaque from baseline [*P* < .001]).

^c^ Two‐sided *P*‐value for between‐treatment difference based on the adjusted mean plaque reduction.

^d^ Per cent treatment difference relative to Paro Junior manual brush (−100 x [treatment difference/ adjusted mean of Paro Junior manual brush]).

**Table 2:** Mean TMQHPI plaque reduction results: mixed dentition for subjects 7 to 9 years of age

Treatment	Baseline mean^a^	Adjusted mean plaque reduction (SE)^b^	Treatment difference (SE)	Treatment difference *P*‐value [90% CI]^c^	Treatment difference (%)^d^
Oral‐B Kids Electric (n = 21)	3.315	0.773 (0.0584)	−0.264 (0.0635)	<.001 (−0.370 to 0.158)	51.9%
Paro Junior Soft Manual (n = 21)	3.259	0.509 (0.0576)			

^a^ Electric and manual brushes did not differ with respect to their baseline (pre‐brushing) plaque levels (2‐sided *P*‐value = 0.233).

^b^ Both electric and manual brushes showed a statistically significant post‐brushing *versus* pre‐brushing plaque reduction when compared to zero (Oral‐B Kids electric brush, 23.5% reduction in plaque from baseline [*P* < .001]; Paro Junior manual brush, 15.5% reduction in plaque from baseline [*P* < .001]).

^c^ Two‐sided *P*‐value for between‐treatment difference based on the adjusted mean plaque reduction.

^d^ Per cent treatment difference relative to Paro Junior manual brush (−100 x [treatment difference/ adjusted mean of Paro Junior manual brush]).

For the primary dentition in the 3‐ to 6‐year age group, both the electric and manual brushes provided statistically significant TMQHPI plaque reductions compared with baseline pre‐brushing (*P* < .001, each). The adjusted whole mouth mean TMQHPI plaque reduction was 0.811 (25.8%) for the electric brush *versus* 0.631 (19.5%) for the manual brush. The treatment difference of − 0.198 was statistically significant (*P* = .005), demonstrating a 32.3% superior plaque removal benefit with the electric brush *versus* the manual brush (Table 1).

For the mixed dentition in the 7‐ to 9‐year age group, both the electric and manual toothbrushes provided statistically significant TMQHPI plaque reductions compared with baseline (*P* < .001, each). The adjusted whole mouth mean TMQHPI plaque reduction was 0.773 (23.5%) for the electric brush *versus* 0.509 (15.5%) for the manual brush. The treatment difference of − 0.264 was statistically significant (*P* < .001), demonstrating a 51.9% superior plaque removal benefit with the electric brush *versus* the manual brush (Table 2).

**Figure 1:**
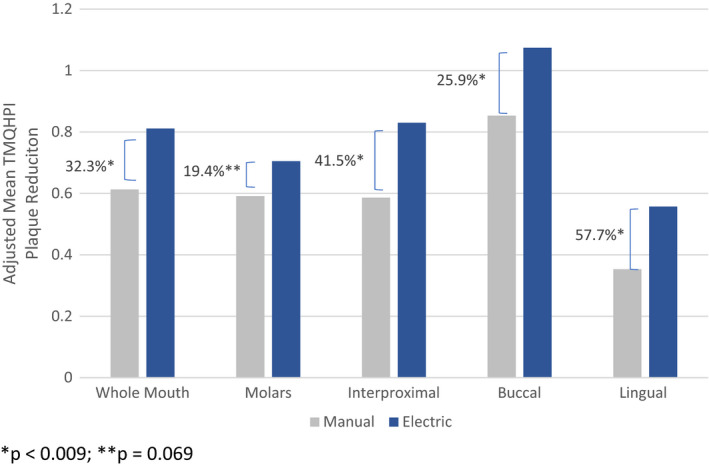
Whole Mouth and Sub‐region Mean TMQHPI plaque reduction results: primary dentition in 3 to 6 year olds

**Figure 2:**
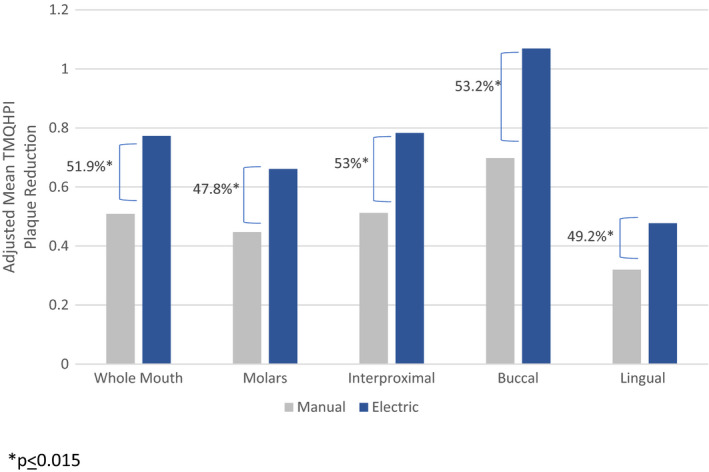
Whole Mouth and Sub‐region Mean TMQHPI plaque reduction results: mixed dentition in 7 to 9 year olds

Sub‐region plaque removal results are shown in Figures 1 and 2. Consistent with whole mouth plaque removal findings, the electric toothbrush provided statistically significantly greater plaque removal for all sub‐regions in both the primary and mixed dentitions (*P* ≤ .015) with the exception of a directional difference for primary molars in the 3‐ to 6‐year‐old group (*P* = .07). The benefit for the electric toothbrush over the manual toothbrush ranged from 19% to 58% for the primary dentition in 3 to 6 year olds and 48% to 53% in the mixed dentition of 7 to 9 year olds.

### 3.3 Safety

Both toothbrushes were well tolerated, with no adverse events reported or observed over the duration of the study.

## 4 DISCUSSION

This randomised, single‐brushing, 2‐treatment, 4‐period crossover study demonstrated statistically significant plaque removal efficacy of an O‐R electric toothbrush compared with a manual brush in the primary dentition of 3‐ to 6‐year‐old children and in the mixed dentition of 7‐ to 9‐year‐old children. The superior plaque removal efficacy of the O‐R toothbrush was seen both in younger children whose parents brushed their teeth for them and in older children who brushed their own teeth, in the challenging environment of the mixed dentition.

Our findings reinforce the importance of toothbrushing to reduce plaque build‐up and prevent the development of caries. Children 7‐9 years of age typically brush their own teeth but are faced with a number of challenges in their daily home brushing routines, including limited dexterity, difficulty establishing and maintaining routines, and lack of patience.^16^ The magnitude of the plaque reduction benefit seen with the electric toothbrush relative to the manual brush for the mixed dentition in the older age group suggests an electric brush is more likely to help children overcome these limitations and achieve a better plaque removal. Similarly, the statistically significant plaque reduction benefit seen with the electric toothbrush *versus* the manual brush in children aged 3‐6 years suggests using an electric brush may help parents overcome some challenges associated with brushing the teeth of their children. In particular, parents brushing the teeth of their children may find an electric toothbrush easier to manoeuvre compared to a manual brush.

To date, limited studies employing different methodologies have evaluated the plaque reduction benefits of electric toothbrushes relative to manual brushes in children; our findings are generally consistent with these previous reports. In 1997, the first study of an electric toothbrush in children to be conducted in over 20 years demonstrated the superior plaque removal efficacy of the Braun Oral‐B Plaque Remover for Kids relative to a manual brush among children 8 to 12 years old.^17^ Another study in children aged 6 to 11 years demonstrated superior plaque reduction with a Braun Oral‐B children's electric toothbrush relative to a manual brush over a 30‐day home use period and in single‐use supervised brushing at baseline.^18^ Previous work from our group in 8 to 11 year olds showed superior plaque removal efficacy with a Braun Oral‐B children's electric toothbrush compared to a manual brush in a randomised, replicate‐use, single‐brushing, crossover clinical trial.^15^

The findings of some other paediatric studies are more mixed. A 3‐year longitudinal study in elementary school children did not find any benefit of an Oral‐B electric toothbrush *versus* a manual brush on reduced caries prevalence.^19^ A study conducted by Silverman and colleagues in 4‐ to 5‐year‐old children compared the plaque removal efficacy of the Oralgiene children's electric toothbrush (an oscillating toothbrush with a unique brush head), the Oral‐B children's oscillating‐rotating electric brush, and a manual brush in both single‐use and 6‐week home use settings. The Oralgiene toothbrush removed significantly more plaque during the single‐use trial and the Braun Oral‐B brush removed significantly more plaque during the 6‐week trial, but no clinically meaningful differences were determined between any of the brushes in terms of plaque removal.^20^ Another study conducted by da Costa et al found a Braun Oral‐B electric toothbrush provided superior plaque removal compared to a manual brush in children aged 4 to 5 years; however, the same result was not seen in children aged 10 to 12 years with mixed dentition, for whom both brushes were deemed equivalent.^21^ Notably, the 4‐ to 5‐year‐old children in both the Silverman and da Costa studies brushed their own teeth,^20^, ^21^ whereas parents brushed the teeth of their 4‐ to 5‐year‐old children in this study.

Oral health habits in children have been shown to establish trajectories that continue into adulthood.^3^, ^4^ The results of our study are consistent with the substantial body of evidence in adult populations demonstrating the significant plaque removal efficacy of electric toothbrushes over manual brushes, with the greatest benefit reported for O‐R electric brushes.^8^, ^9^ In addition, a recent 11‐year cohort study assessing electric *versus* manual brush use in adults suggests that the advantages seen with electric brushes likely translate into substantial benefits over time including reduced progression of decayed, missing and filled surfaces (DMFS) and clinical attachment loss, both of which have been found to translate into retaining more number of teeth.^22^

There are some limitations to this research. This study was based on a single‐brushing exercise, so a longer‐term clinical study should be considered in the future to confirm these findings in a paediatric population. The replicate‐use design used, which ensured each child used both brushes twice during the course of the 4‐period study, has been shown to corroborate longer‐term results in O‐R toothbrushing studies of adults.^6^, ^8^ Additional research in other paediatric populations, including those with different baseline plaque levels and/or in different socioeconomic groups, is recommended to determine if those factors impact outcomes.

In conclusion, an O‐R electric toothbrush provided superior plaque reduction relative to a manual brush with single‐use brushing in the primary dentition of children aged 3‐6 years whose parents brushed their teeth and in the mixed dentition of children aged 7‐9 years who brushed their own teeth.

## AUTHOR CONTRIBUTIONS

ED, AZ, JG, RCV developed the protocol; ED, AZ, ED, HT contributed to the study execution; ED, AZ were the investigators; JG analysed the data; all authors interpreted the data; and all authors reviewed and approved the manuscript.

## CONFLICT OF INTEREST

Dr. Ccahuana‐Vasquez, Dr. Timm, and Dr. Grender are employees of Procter & Gamble. Dr. Davidovich and Dr. Zini have received grants from Procter & Gamble.
